# Thermodynamic Modeling and Performance Analysis of Vehicular High-Temperature Proton Exchange Membrane Fuel Cell System

**DOI:** 10.3390/membranes12010072

**Published:** 2022-01-05

**Authors:** Yanju Li, Dongxu Li, Zheshu Ma, Meng Zheng, Zhanghao Lu

**Affiliations:** College of Automobile and Traffic Engineering, Nanjing Forestry University, Nanjing 210037, China; liyanju@njfu.edu.cn (Y.L.); lidongxu@njfu.edu.cn (D.L.); zhengmeng@njfu.edu.cn (M.Z.); luzhanghao@njfu.edu.cn (Z.L.)

**Keywords:** HT-PEMFC, thermodynamic modeling, powertrain design, exergy analysis, energy analysis

## Abstract

Since the high temperature proton exchange membrane fuel cells (HT-PEMFC) stack require a range of auxiliary equipments to maintain operating conditions, it is necessary to consider operation of related components in the design of HT-PEMFC systems. In this paper, a thermodynamic model of a vehicular HT-PEMFC system using phosphoric acid doped polybenzimidazole membrane is developed. The power distribution and exergy loss of each component are derived according to thermodynamic analysis, where the stack and heat exchanger are the two components with the greatest exergy loss. In addition, ecological functions and improvement potentials are proposed to evaluate the system performance better. On this basis, the effects of stack inlet temperature, pressure, and stoichiometric on system performance are analyzed. The results showed that the energy efficiency, exergy efficiency and net output power of the system achieved the maximum when the inlet gases temperature is 406.1 K. The system performance is better when the cathode inlet pressure is relatively low and the anode inlet pressure is relatively high. Moreover, the stoichiometry should be reduced to improve the system output performance on the basis of ensuring sufficient gases reaction in the stack.

## 1. Introduction

Recently, the demand for energy-efficient and eco-friendly energy systems has been increasing with the growing problems such as depletion of fossil fuels and environmental deterioration [1,2,3,4,5,6,7]. Proton exchange membrane fuel cells (PEMFC) with benefits of zero-emission, high energy conversion efficiency, high power density and low maintenance are widely used in fuel cell vehicles (FCVs) [8,9,10,11,12]. Perfluorosulfonic acid (Nafion) is commonly used as a membrane in low temperature proton exchange membrane fuel cells (LT-PEMFCs) [13,14,15,16]. Since the proton conductivity inside the Nafion membrane requires water as a charge carrier, the membrane must always be kept in a hydrated state to maintain optimal performance [17,18]. Compared to LT-PEMFC, HT-PEMFC simplifies water and heat management system and accelerates reaction kinetic at the electrode [19]. Higher operating temperatures can also improve CO tolerance and improve the quality of waste heat [20,21,22]. In recent years, a range of plasmonic conductor polymer membranes have been developed and modified which enable operation in the higher temperature 120–200 °C [23,24,25,26,27,28]. Phosphoric-acid-doped polybenzimidazole (PA/PBI) membranes are widely used in HT-PEMFC due to its excellent mechanical strength and good chemical resistance [29,30,31]. In fact, polybenzimidazole (PBI) is an amorphous rigid polymer doped with phosphoric acid [32,33]. Compared to other acids at high temperatures, phosphoric acid has low vapor pressure and high thermal stability [34,35,36,37,38,39]. In this paper, a thermodynamic model of the vehicular HT-PEMFC system was established based on a PBI membrane.

From a thermodynamic point of view, a complete vehicular HT-PEMFC system consists of HT-PEMFC stack, hydrogen supply sub-system, air supply sub-system and a thermal management sub-system [40,41]. HT-PEMFC single cell is the main core component in the stack and its performance has a great impact on the overall system operation. Guo et al. [42] developed a thermodynamic model of a HT-PEMFC single cell and studied the effect of the main operating conditions and designing parameters on the performance of the HT-PEMFC sing cell. The results showed that higher operating temperatures and operating pressures can effectively improve the output performance of HT-PEMFC single cell. However, the power distribution of the ancillary equipment that maintains these operating conditions is not considered. Qin et al. [43] properly equipped an air compressor with the PEMFC stack, and optimized the operating pressure of the power system. The results showed that both the power generation of the fuel cell stack and the power consumption of the compressor increase with the system operating pressure. Zhang et al. [44] developed a simulation model of PEMFC system with hydrogen cycle and dead-ended anode. The results showed that the hydrogen cycle fuel cell system with dead-end anodes had good performance and the control strategy was effective. Reddy et al. [45] investigated the effectiveness of the HT-PEMFC external coolant system by using a multi-scale stacked heat transfer model. The simulation results showed that the temperature variation in the stack could be kept within 10 K by optimizing the number of cooling plates, the coolant flow rate and the temperature entering the stack. Most of these studies focused on the analysis of fuel cell system components, such as HT-PEMFC stack [46], air compressor [47], hydrogen circulation pump [48] and combinations of very few components of the system [49,50], lacking overall system modeling and performance analysis.

At present, in addition to the research on fuel cell system components, there are some studies on the overall PEMFC system. Chen et al. [51] established a PEMFC system thermodynamic model and applied a novel multi-objective evolutionary algorithm based on decomposition (MOEA/D) to optimize the operating parameters of the PEMFC system in order to maximize system efficiency and power. The final optimized point of system energy efficiency and electrical power can reach 79% and 8.04 kW, respectively. However, the components of the PEMFC system and their connections were not described in detail. Chitsaz et al. [52] presented the layout structure of the PEMFC system layout, and its thermodynamic and the exergoeconomic assessment was carried out. In addition, the effects of current density and temperature on the performance of the PEMFC system were investigated. Hwang et al. [53] developed a PEMFC cogeneration system that provided high quality electricity and hot water. The results showed that the maximum system efficiency was as high as 81% when combining heat and power. Mert et al. [54] dealt with the exergoeconomic analysis of a vehicular PEMFC system. It was found that with the temperature and pressure increased and the membrane thickness decreased, the system efficiency increased, leading to a reduction in overall production cost. Blum et al. [55] elaborated the system layout concepts of PEMFC, phosphoric acid fuel cell (PAFC) and solid oxide fuel cell (SOFC), and analyzed their electrical efficiencies. The results show that the electrical efficiency of different types of fuel cell systems varies greatly.

Based on the above research background, a complete thermodynamic model of the vehicular HT-PEMFC system is developed in this paper to provide a reference for the future design and optimization of the fuel cell system. The model fully takes the power distribution of each component and their connection methods into account. Energy and exergy distribution based on thermodynamic model are revealed, and the evaluation method of the system is established. In addition, ecological functions and improvement potentials are derived to evaluate the system performance. Then, the future optimization of the system is suggested based on the effect of different parameters on the system performance. The rest of this paper is organized as follows: The schematic and description of the proposed vehicular HT-PEMFC system is given in Section 2. In Section 3, the thermodynamic model of the system is given, including the HT-PEMFC stack and other auxiliary equipment. The power consumption and exergy distribution of different components are determined by energy analysis and energy analysis. Section 4 analyzes the effect of stack inlet temperature, pressure and stoichiometry on system performance. Contents of this paper are concluded in Section 5.

## 2. System Description

**Figure 1:**
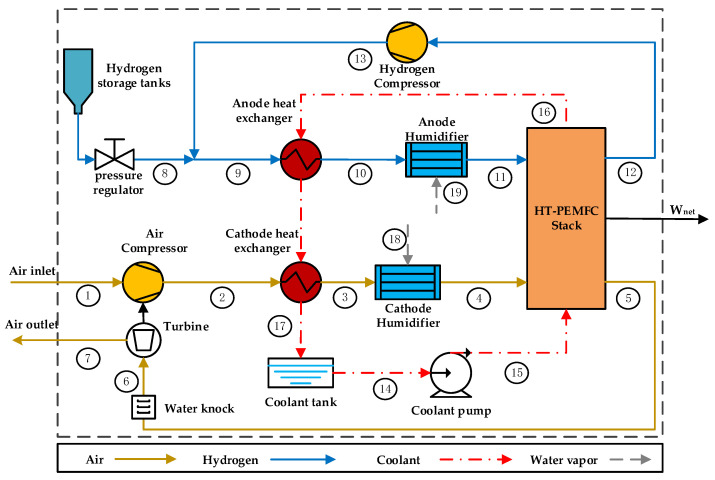
Schematic of the proposed HT-PEMFC system.

In order to meet the operating conditions of the HT-PEMFC stack, the air from the environment and hydrogen from the hydrogen storage tank should be pressurized, heated and humidified before entering the stack, and the waste heat from the stack should be removed. Therefore, the vehicular HT-PEMFC system should include: HT-PEMFC stack, compressors, humidifiers, heat exchangers and a coolant pump. Meanwhile, in order to make full use of the energy in the cathode exhaust gas, a turbine is installed at the cathode outlet. The use of turbine can effectively reduce the power consumption of the compressor as well as improve the energy utilization and overall efficiency of the system. The schematic of the vehicular HT-PEMFC system developed in this paper is shown in Figure 1. The arrows in Figure 1 indicate the direction of fluid flow in the system, and the numbers indicate the state of the fluid.

The yellow line in Figure 1 indicates the gas supply sub-system, which uses an exhaust gas energy recovery strategy [56]. It mainly consists of air compressor (AC), cathode heat exchanger (CHE), cathode humidifier (CH) and turbine (TUR). The air in the environment is first pressurized by an air compressor to the required operating pressure of the stack. Then, the compressed air is preheated by the high temperature coolant from the stack. The air needs to be humidified before entering the stack to improve the proton conductivity of the PEM. This system uses external spray humidification method. Finally, the incompletely reacted exhaust gas from the environment and water vapor are discharged from the cathode outlet of the stack and then flow into the turbine for energy recovery before being discharged into the environment. The blue line in Figure 1 is the hydrogen supply sub-system, which uses a hydrogen recirculation strategy [57]. It mainly consists of a hydrogen compressor (HC), an anode heat exchanger (AHE) and an anode humidifier (AH). The hydrogen from the hydrogen storage tank is passed through a pressure regulator to reach the pressure required for HT-PEMFC stack operation and then is mixed with hydrogen from hydrogen compressor. The mixed hydrogen gas flows into the HT-PEMFC stack after heating and humidifying. The incompletely reacted hydrogen is recycled by a hydrogen compressor. The red line in Figure 1 is the thermal management sub-system. It consists of a coolant pump (CP), AHE and CHE. The coolant for HT-PEMFC system is Tri-ethylene glycol (TEG) because its phase does not change at 423~463 K [58,59]. The coolant carries heat away from the stack to preheat the inlet gases through the heat exchangers.

In order to simplify the thermodynamic model, several reasonable assumptions are made:

(1) The system works under steady-state conditions, and the heat loss and pressure of the system loss are neglected [60,61].
(2) The ambient air temperature is 298.15 K, the pressure is 101 kPa [60].
(3) Dry air and hydrogen behave as ideal gas. Hydrogen is 100% pure and reacts completely in the fuel cell. Air is composed of 21% oxygen and 79% nitrogen [62].
(4) The working temperature of the stack is uniform. The temperature rise of both gases and coolant in the stack is fixed at 10 K, and the pressure drop is fixed at 0.2 atm [60].
(5) Energy loss and exergy losses during the gas flow are not considered [63].
(6) All the heat generated by the stack is carried away by the coolant [63].

## 3. Thermodynamic Modeling and Analysis

### 3.1. HT-PEMFC Stack

The HT-PEMFC stack model developed in this paper is based on HT-PEMFC single cell using PBI membranes, which have been validated in our previous studies [64,65,66]. The proton conduction mechanism of the PA/PBI membrane is called “Grotthus mechanism” [67]. The electrochemical reactions within HT-PEMFC be described as [68]:

(1) $$\mathrm{Anode}:\;H_{2}\to 2H^{+}+2e^{-}\;$$

(2) $$\mathrm{Cathode}:\;2H^{+}+\frac{1}{2}O_{2}+2e^{-}\to H_{2}O$$

(3) $$\mathrm{Overall}\;\mathrm{recation}:H_{2}\left(g\right)+\frac{1}{2}O_{2}\left(g\right)\to H_{2}O\left(g\right)+\mathrm{heat}+\mathrm{electricity}$$

The reversible output voltage of a HT-PEMFC single cell is expressed:

(4) $$U_{cell}=E_{rev}-E_{act}-E_{ohm}-E_{con}=E_{rev}-\left(1+\frac{1}{\alpha }\right)\frac{RT}{n_{e}}\ln\left(\frac{J_{L}}{J_{L}-J}\right)-\frac{RT}{n_{e}\alpha F}\ln\left(\frac{J+J_{leak}}{J_{0}}\right)-J\left(\frac{t_{mem}}{\sigma_{mem}}\right)$$

where $U_{cell}$ is the output voltage of a HT-PEMFC single cell. $E_{rev}$ is the reversible cell voltage that can be calculated from the Nernst equation. $E_{act}$, $E_{ohm}$ and $E_{con}$ represent the activation overpotential, ohmic overpotential and concentration overpotential, respectively. α is the charge transfer coefficient. J is the operating current density. $J_{L}$ is the limiting current density. $J_{0}$ and $J_{leak}$ are the exchange current density and leak curent density, respectively. $t_{mem}$ and $\sigma_{mem}$ are the thickness and proton conductivity of the membrane, respectively [64]. $\sigma_{mem}$ can be calculated by:

(5) $$\sigma_{mem}=\frac{ab}{T}e^{\frac{-c_{act}}{RT}}\;$$

(6) $$a=68DL^{3}-6324DL^{2}+65750DL+8460$$

(7) $$b=\{\begin{array}{c} 1+\left(0.01704T-4.767\right)RH\;373.15K\leq T\leq 413.15\\ 1+\left(0.1432T-56.89\right)RH\;\;413.15K<T\leq 453.15\\ 1+\left(0.7T-309.2\right)RH\;\;\;\;\;\;\;\;\;453.15<T\leq 473.15 \end{array}$$

(8) $$c_{act}=-619.6DL+21750$$

where DL is the doping level of PA. RH is the relative humidity of the electrolyte. Kim et al. [69] suggested empirical model explaining that PA doping level is dropped from initial doping level depending on time t. DL can be calculated by:

(9) $$DL=DL_{0}-DL_{DC}\cdot t$$

where $DL_{0}$ is initial doping level, $DL_{DC}$ is doping level drop coefficient.

**Figure 2:**
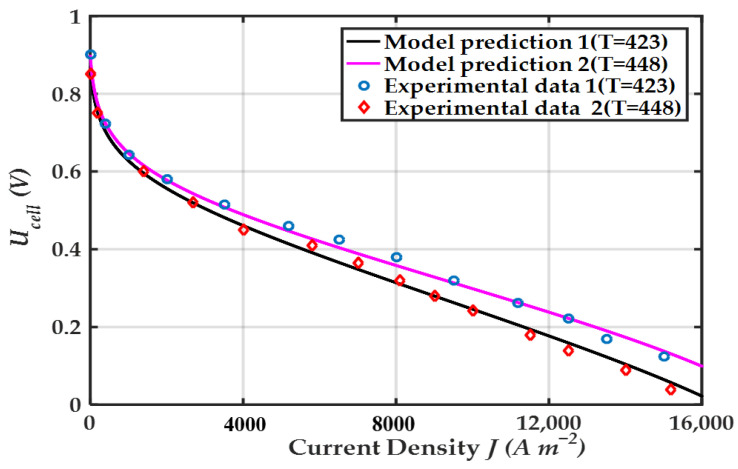
Comparisons of the HT-PEMFC single cell output voltage between modeling results and the experimental data.

**Figure 3:**
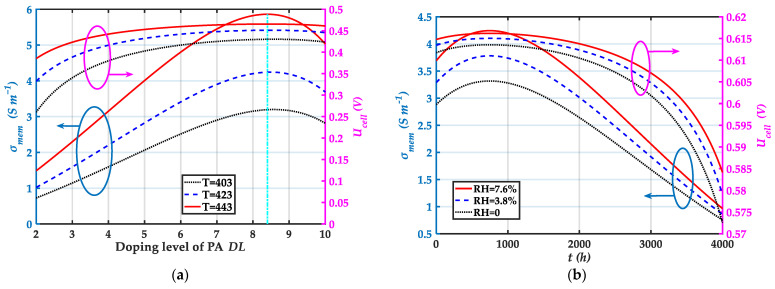
Variation of proton conductivity of the membrane $\sigma_{mem}$ and reversible output voltage of the HT-PEMFC single cell $U_{cell}$ with DL and t: (**a**) Variation of $\sigma_{mem}$ and $U_{cell}$ with DL; (**b**) Variation of $\sigma_{mem}$ and $U_{cell}$ with t.

Figure 2 is a comparison of model prediction and experimental data from ref. [70]. The results show that the model is in good agreement with the experimental data. The variation of proton conductivity of the membrane $\sigma_{mem}$ and reversible output voltage of the HT-PEMFC single cell $U_{cell}$ with DL and t at current density $J=0.8\;{A\;\mathrm{cm}}^{-2}$ is given in Figure 3. Figure 3a shows that $\sigma_{mem}$ and $U_{cell}$ achieve the maximum value when DL=8.4. A higher degree of phosphoric acid doping is beneficial to improve the HT-PEMFC performance, but too high DL also results in a poorer mechanical property and makes phosphoric acid molecules more easily to leak out of the HT-PEMFC [71]. As the temperature increases, both $\sigma_{mem}$ and $U_{cell}$ increase. The increase in temperature facilitates the reduction of internal resistance, thus improving the single cell output performance. From Figure 3b, $\sigma_{mem}$ and $U_{cell}$ first increases and then gradually decreases with time. In the lifetime test of HT-PEMFC, there was an activation stage at the beginning that led to improved performance [72]. After that, the phosphoric acid in the PBI membrane continues to be lost, $\sigma_{mem}$ and $U_{cell}$ gradually decrease. Therefore, the leakage of PA is one of the main reasons for the durability of PA doped PBI membranes and is one of the urgent problems that need to solve at present. In addition, the increase of electrode relative humidity is beneficial for proton conductivity and single cell output voltage.

The electric power and thermal power output of the HT-PEMFC stack is expressed as:

(10) $$W_{stack}=N\cdot U_{cell}\cdot J\cdot A\;$$

(11) $$Q_{stack}=N\cdot \left(E_{rev}-U_{cell}\right)\cdot J\cdot A$$

where $W_{stack}$ is the electric power output of the stack, $Q_{stack}$ is the thermal power. N is the number of HT-PEMFC single cells, and A is the effective working area of a single cell.

This paper provides a power system design for FCVs based on the above HT-PEMFC single cell model, and uses mathematical equations to build the HT-PEMFC system model by Matlab programming. The operating and design parameters of the vehicular HT-PEMFC system model in this paper are given in Table 1. The design parameters of HT-PEMFC single cell can be found in ref. [50].

### 3.2. Auxiliary Equipment

The inlet and outlet gas flow rates of the fuel cell stack should be determined before the auxiliary equipment is modeled. According to the above reaction Equation (1), it is known that 1 mol of hydrogen can transfer 2 mol of electrons, therefore, the mass flow rate of hydrogen at the stack inlet can be expressed as [73]:

(12) $${\dot{m}}_{H_{2},in}=S_{an}\cdot M_{H_{2}}\cdot \frac{N\cdot J}{2F}=S_{an}\cdot M_{H_{2}}\cdot \frac{N\cdot J\cdot A}{2F}\;$$

where F is the Faraday constant, $S_{an}$ is the anode stoichiometric ratio.

Similarly, the air mass flow rate at the stack inlet can be obtained as [73]:

(13) $${\dot{m}}_{air,in}=S_{ca}\cdot M_{O_{2}}\cdot \frac{N\cdot I}{4F\cdot g_{O_{2}}}=S_{ca}\cdot M_{O_{2}}\cdot \frac{N\cdot J\cdot A}{4F\cdot g_{O_{2}}}\;$$

where $S_{ca}$ is the anode stoichiometric ratio, and $g_{O_{2}}$ represents the oxygen mass fraction in the air.

The gas at the cathode and anode inlets should have a certain humidity to ensure more efficient operation of the HT-PEMFC stack. The ratio of the mass of water vapor to the mass of dry gas is called the moisture content in the wet gas, and it can be obtained [63]:

(14) $$d=\frac{m_{v}}{m_{a}}=\frac{M_{v}}{M_{a}}\frac{RH\cdot p_{s}}{p_{wet}-RH\cdot p_{s}}\;$$

where $m_{v}$ and $m_{a}$ are the masses of water vapor and dry gas, respectively. $M_{v}$ and $M_{a}$ are the relative molar masses of water vapor and dry gas, respectively. $p_{wet}$ is the pressure of wet gas. $p_{s}$ is the saturated vapor pressure. Thus, the mass flow rate of water vapor can be obtained [63]:

(15) $${\dot{m}}_{v}={\dot{m}}_{a}\cdot d\;$$

The mass flow rates at the cathode and anode of the stack inlet can be expressed as:

(16) $${\dot{m}}_{4}={\dot{m}}_{air,ca}+{\dot{m}}_{v,ca}\;$$

(17) $${\dot{m}}_{11}={\dot{m}}_{H_{2},an}+{\dot{m}}_{v,an}$$

where ${\dot{m}}_{v,ca}$ and ${\dot{m}}_{v,an}$ are the mass flow rates of water vapor in the wet gas at the cathode and anode of the stack inlet, respectively. The numbers in the subscripts correspond to the state of the fluid in Figure 1.

The mass flow rates at the cathode and anode of the stack outlet can be calculated by applying the mass balance equations:

(18) $${\dot{m}}_{5}={\dot{m}}_{4}-M_{O_{2}}\cdot \frac{N\cdot J\cdot A}{4F}$$

(19) $${\dot{m}}_{12}={\dot{m}}_{11}-M_{H_{2}}\cdot \frac{N\cdot J\cdot A}{2F}$$

#### 3.2.1. Compressors

The compressor can pressurize the inlet air and the incompletely reacted hydrogen to the working pressure of the stack. The power consumed by the air compressor and hydrogen compressor can be expressed as:

(20) $$W_{AC}=\frac{C_{p,1}{\dot{m}}_{1}T_{1}}{\eta_{C}}\left({\left(\frac{p_{2}}{p_{1}}\right)}^{\frac{\gamma -1}{\gamma }}-1\right)$$

(21) $$W_{HC}=\frac{C_{p,12}{\dot{m}}_{12}T_{12}}{\eta_{C}}\left({\left(\frac{p_{13}}{p_{12}}\right)}^{\frac{\gamma -1}{\gamma }}-1\right)$$

where $W_{AC}$ and $W_{HC}$ represent the power consumed by the air and hydrogen compressors, respectively. $C_{p}$ is the specific heat at constant pressure, γ is the adiabatic coefficient. p and T represent the temperature and pressure in different state, respectively.

The temperature of the compressor outlet can be obtained:

(22) $$T_{2}=T_{1}\cdot \left({\left(\frac{p_{2}}{p_{1}}\right)}^{\frac{\gamma -1}{\gamma }}-1\right)$$

(23) $$T_{13}=T_{12}\cdot \left({\left(\frac{p_{13}}{p_{12}}\right)}^{\frac{\gamma -1}{\gamma }}-1\right)\;$$

#### 3.2.2. Humidifiers

The humidification process is considered as an adiabatic process, which satisfies the law of conservation of energy. Thus, the cathode and anode humidification process can be expressed as:

(24) $$C_{p,4}{\dot{m}}_{4}T_{4}=C_{p,3}{\dot{m}}_{3}T_{3}+C_{p,9}{\dot{m}}_{9}T_{9}\;$$

(25) $$C_{p,11}{\dot{m}}_{11}T_{11}=C_{p,10}{\dot{m}}_{10}T_{10}+C_{p,20}{\dot{m}}_{20}T_{20}\;$$

The process of gas 8 and gas 13 to gas 9 in Figure 1 is also consistent with the energy conservation theorem, and the process can be expressed as:

(26) $$C_{p,9}{\dot{m}}_{9}T_{9}=C_{p,8}{\dot{m}}_{8}T_{8}+C_{p,13}{\dot{m}}_{13}T_{13}\;$$

#### 3.2.3. Heat Exchangers

When the heat exchangers heat the inlet gas, part of the heat comes from the coolant through heat exchange and the other part is provided by the electric heater. It is considered that the heat required for gas 2 and gas 9 to reach $T_{15}$ is provided by the coolant, which rejects heat at the heat exchanger [63]. However, the heating from state 15 to state 10 and state 3 needs to be heated by electric heaters, respectively. Thus, the heating power of the heat exchanger of the cathode and anode is:

(27) $$W_{CHE}=C_{p,2}{\dot{m}}_{2}\left(T_{3}-T_{15}\right)\;$$

(28) $$W_{AHE}=C_{p,2}{\dot{m}}_{2}\left(T_{10}-T_{15}\right)$$

#### 3.2.4. Coolant Pump

The coolant pump extracts coolant into the fuel cell stack to remove excess heat, which is used in the heat exchanger for heating. The coolant flow rate can be obtained by energy conservation. The coolant flow rate is:

(29) $${\dot{m}}_{15}=\frac{Q_{stack}}{C_{p,15}\left(T_{16}-T_{15}\right)}$$

The power consumed by the coolant pump is expressed as:

(30) $$W_{CP}=\frac{{\dot{m}}_{15}\cdot \left(p_{16}-p_{15}\right)}{\rho_{CP}\cdot \eta_{CP}}\;$$

where $\rho_{CP}$ is the density of the coolant, $\eta_{CP}$ is the efficiency of the coolant pump.

#### 3.2.5. Turbine

The use of turbine can effectively reduce the power loss of air compressor and improve the energy utilization. The turbine works just the opposite of the air compressor, and its output power is:

(31) $$W_{TUR}=C_{p,6}\cdot {\dot{m}}_{6}\cdot T_{6}\cdot \eta_{TUR}\left(1-{\left(\frac{P_{7}}{P_{6}}\right)}^{\frac{\gamma -1}{\gamma }}\right)\;$$

According to the above model, the power of each component at different current densities can be obtained, as shown in Table 2. Positive values represent the power output and negative values represent the power consumed. From Table 2, when the current density increases, the output power of the HT-PEMFC stack increases, but the power consumption of the ancillary equipment also increases. The reason for this is that increasing current density leads to an increase in the flow rate of the fluid, thus increasing the power consumption of the ancillary equipment. By comparison, it can be found that AC is the most consuming component of the ancillary equipment. In order to reduce parasitic power consumption and further improve the efficiency of the system, the design and operation of the AC should be carefully considered.

### 3.3. Thermodynamic Analysis

In order to better evaluate the thermodynamic performance of vehicular HT-PEMFC systems, the energy analysis and the exergy analysis of the system are performed according to the first and second laws of thermodynamics, respectively.

#### 3.3.1. Energy Efficiency

The power consumed by the auxiliary equipment is:

(32) $$W_{comsume}=W_{AC}+W_{HC}+W_{CHE}+W_{AHE}+W_{CP}$$

The net output power of the HT-PEMFC power system can be expressed as:

(33) $$W_{net}=W_{stack}-W_{consume}+W_{TUR}$$

The energy efficiency of the system is:

(34) $$\eta_{energy}=\frac{W_{net}\cdot U_{cell}}{W_{stack}\cdot E_{rev}}$$

#### 3.3.2. Exergy Efficiency

Exergy is the portion of energy that can be converted into useful work during a fully reversible change in the environment [74,75]. Unlike energy analysis, exergy analysis takes into account the energy limitations, losses and conversion efficiency of the system to evaluate the performance of the system [76,77]. Exergy is mainly divided into mass flow exergy and energy flow exergy.

Mass flow exergy consists mainly of the physical exergy and the chemical exergy. thus mass flow exergy $E_{x}$ can be expressed as [21]:

(35) $$E_{x}=e\cdot \dot{m}=(e^{ph}+e^{ch})\cdot \dot{m}$$

where e is the specific exergy of fluid. $e^{ph}$ and $e^{ch}$ are the specific physical exergy and the specific chemical energy, respectively. $e^{ph}$ and $e^{ch}$ can be expressed as [78]:

(36) $$e^{ph}=\sum \left(h_{i}-h_{0}\right)-T_{0}\cdot \left(s_{i}-s_{0}\right)$$

(37) $$e^{ch}=\left(\sum x_{i}e_{i}^{ch}+R\cdot T_{0}\cdot \sum x_{i}lnx_{i}\right)$$

where h and s are the specific enthalpy and entropy of substances, respectively. $T_{0}$ is the reference temperature. $e_{i}^{ch}$ is the specific chemical exergy of the substances. The standard chemical exergy of several substances could be found in ref. [60].

Energy flow exergy consists mainly of exergy of work and exergy of heat. exergy of work $E_{x,W}$ and exergy of heat $E_{x,Q}$ can be expressed as:

(38) $$E_{x,W}=W$$

(39) $$E_{x,Q}=W_{Q}=\left(1-\frac{T}{T_{0}}\right)Q$$

The exergy balance equation of each component can be expressed as [63]:

(40) $$E_{x,in}=E_{x,out}+W_{output}+E_{x,heat}+E_{x,loss}$$

where $E_{x,in}$ and $E_{x,out}$ indicate the mass flow energy of the input and output components. $W_{output}$ and $E_{x,heat}$ are the output power of the components and the exergy of heat produced, respectively. $E_{x,loss}$ is the exergy loss of the component.

The exergy efficiency of a system is defined as the ratio of the net output power of the system to the input energy of the system, and can be expressed as:

(41) $$\eta_{exergy}=\frac{W_{net}}{E_{x,in,sys}}$$

where $E_{x,in,sys}$ is the exergy of input hydrogen.

**Figure 4:**
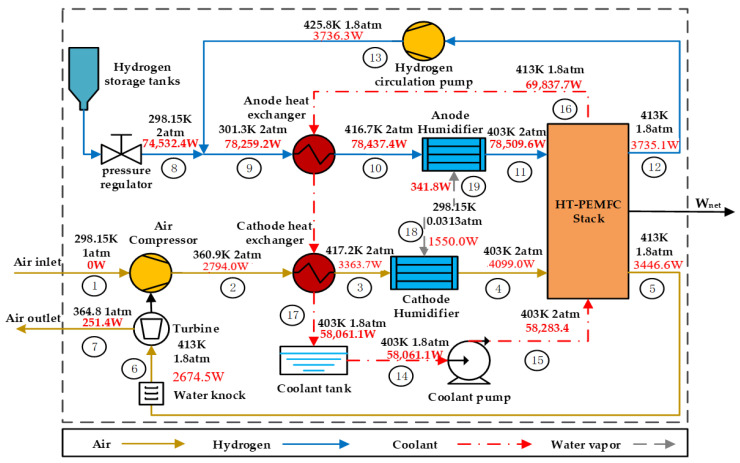
Temperature, pressure and mass flow exergy distribution in the HT-PEMFC system at current density J=0.8 A cm^2^.

The temperature, pressure and mass flow exergy distribution at current density $\;J=0.8\;{A\;\mathrm{cm}}^{-2}$ can be obtained by thermodynamic analysis, as shown in Figure 2. The black font represents the temperature and pressure of the fluid, and the red font represents the mass flow exergy of the fluid. The exergy distribution in the system is intuitively seen in the Figure 4. As shown in Table 3, the exergy loss caused by each component or thermal processes in the system can be obtained according to the exergy balance equation. From Table 3, the energy loss caused by the heat exchanger is the largest among the ancillary equipment, which is mainly because the large amount of heat absorbed by the coolant that is not fully utilized and dissipated into the environment. Therefore, it is significant to find an effective method to recover the waste heat from the stack [56].

From Table 2, it can be found that when the current density is 0.8 ${A\;\mathrm{cm}}^{-2}$, the net output power of the system is 24,877.1 W. At this time, the energy efficiency and exergy efficiency of the system are 35.3% and 33.4%. As a result, the energy conversion efficiency at the HT-PEMFC stack should be improved as much as possible to reduce the exergy loss there. In addition, the turbine can produce 1887.9 W of energy and improve the energy efficiency of the system by 2.67%. Therefore, the use of turbines can effectively reduce the consumption of air compressors and improve system efficiency. It is worth noting that if the system does not use coolant to heat the inlet gas, CHE and AHE will consume 2440.4 W and 526.4 W of heating power at current density $J=0.8\;\;{A\;\mathrm{cm}}^{-2}$, respectively. Therefore, using coolant to heat the inlet gases can save 2221.2 W of energy and increase the system efficiency by 3.15%.

#### 3.3.3. Ecological Function

Angulo-brown [79] derived the concept of ecological function according to Newton’s heat transfer law when studying heat engines. The ecological function is the difference between the net output power of the system and the exergy loss. It is a new performance indicator that optimizes the tradeoff between the output power and the entropy production, aiming to improve output power and reduce exergy loss at the same time. The ecological function E is expressed as:

(42) $$E=W_{net}-E_{x,loss,sys}$$

where $E_{x,loss,sys}$ is the total exergy loss of the system.

#### 3.3.4. Improvement Potential

The improvement potential is a metric proposed based on exergy efficiency and indicates the room for improvement in system performance [80]. The improvement potential IP is expressed as:

(43) $$IP=\left(1-\eta_{exergy,sys}\right)\cdot \left(E_{x,in,sys}-E_{x,out,sys}\right)$$

**Figure 5:**
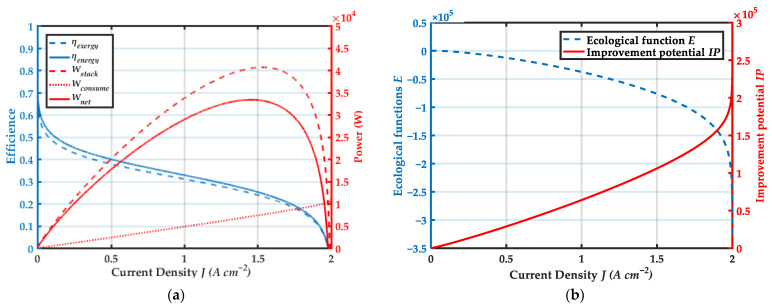
Variation trends of system performance with current density: (**a**) Variation trends of $\eta_{energy}$, $\eta_{exergy}$, $W_{stack}$, $W_{comsume}$ and $W_{net}$ with current density J; (**b**) Variation trends of ecological function E and improvement potential IP with current density J.

Figure 5 shows the variation trends of the system performance with current density are obtained. From Figure 5a, it can be seen that both energy efficiency and exergy efficiency decrease with the increase of current density. As current density increases, the power consumed by ancillary equipment increases. This is mainly because flow rate accelerates with the increase of current density, resulting in the improve of auxiliary equipment power. From Figure 5b, it can be seen that the ecological function E gradually decreases and the improvement potential IP gradually increases with the increase of current density. It shows that the ecological performance is better in the low current density region, and the exergy loss is relatively small. There is more room for improvement in the high current density region to improve the output performance of the system. Due to the limited space available for vehicle powertrain installation, it is necessary to achieve high power density in high current density operation mode [81,82,83,84].

## 4. Results and Discussion

Different operating parameters of the system have a significant impact on the system performance [51]. In this paper, the effects of stack inlet temperature, inlet pressure, and inlet stoichiometry on the system output performance are studied for vehicular HT-PEMFC stacks.

### 4.1. Effect of Stack Inlet Temperature

**Figure 6:**
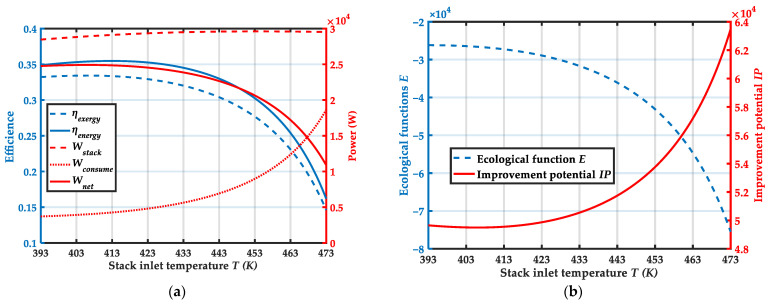
Variation trends of system performance with stack inlet temperature T: (**a**) Variation trends of $\eta_{energy}$, $\eta_{exergy}$, $W_{stack}$, $W_{comsume}$ and $W_{net}$ with stack inlet temperature T; (**b**) Variation of ecological function E and improvement potential IP with stack inlet temperature T.

Figure 6 shows variation trends of system performance with stack inlet temperature T. From Figure 6a, the energy efficiency, energy efficiency and net output power of system have a small increase and then gradually decrease as the stack inlet temperature increases. When the inlet temperature of the stack T = 406.1 K, the net output power of the system achieves the maximum value, the net output power of the system, the energy efficiency and energy efficiency are 24,907.4 W, 35.4% and 33.4% respectively. This is due to when the stack inlet temperature increases, the increase in auxiliary equipment power consumption is greater than the increase in stack power generation. From Figure 6b, the ecological performance of system is poor when the stack inlet temperature is too high, and the potential for improvement is large. Therefore, if the stack inlet temperature is too high, the system output performance will become worse.

### 4.2. Effect of Cathode and Anode Inlet Pressures

**Figure 7:**
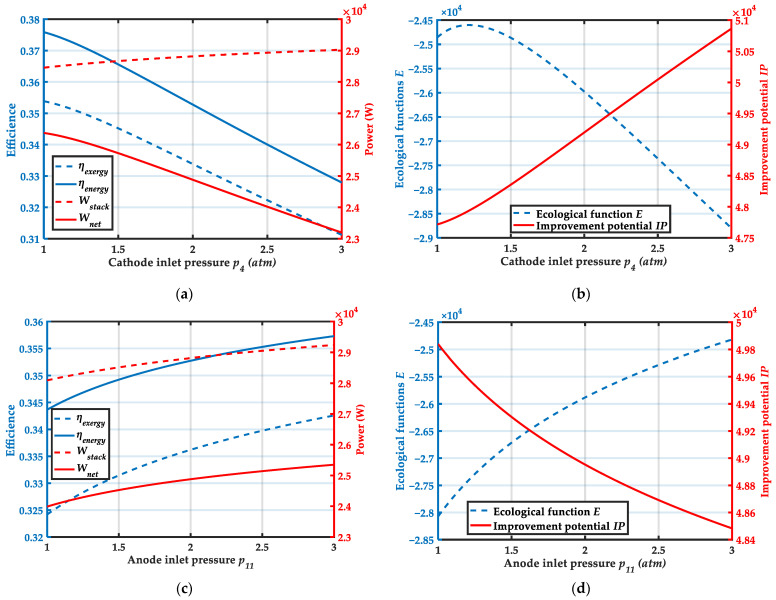
Variation trends of system performance with cathode and anode inlet pressures: (**a**) Variation trends of $\eta_{energy}$, $\eta_{exergy}$, $W_{stack}$ and $W_{net}$ with cathode inlet pressures $p_{4}$; (**b**) Variation trends of ecological function E and improvement potential IP with cathode inlet pressures $p_{4}$. (**c**) Variation trends of $\eta_{energy}$, $\eta_{exergy}$, $W_{stack}$ and $W_{net}$ with anode inlet pressures $p_{11}$; (**d**) Variation trends of ecological function E and improvement potential IP with anode inlet pressures $p_{11}$.

Figure 7a,b show the variation of system performance with cathode inlet pressure. From Figure 7a, when the cathode inlet pressure increases from 1 atm to 3 atm, the output power of the HT-PEMFC stack continuously increases, but $\eta_{energy}$, $\eta_{exergy}$ and $W_{net}$ all decrease. The energy efficiency and exergy efficiency of the system decreased from 37.6% and 35.4%, respectively. The net output power of the system decreases from 26,373.3 W to 23,195.9 W. This is mainly due to the large increase in power consumption of the air compressor when the cathode inlet pressure increases. From Figure 7b, it is found that the ecological function of the system attains the maximum value when the cathode inlet pressure is 1.2 atm. The improvement potential increases with increasing cathode inlet pressure. This means that the system has less room for improvement at lower cathode inlet pressures. Therefore, the system output performance is better when the cathode inlet pressure is lower.

Figure 7c,d show the variation of system performance with anode inlet pressure. From Figure 7c, $\eta_{energy}$, $\eta_{exergy}$, $W_{stack}$ and $W_{net}$ increase constantly as the anode inlet pressure increases. When the anode pressure increased from 1 atm to 3 atm, the energy efficiency and exergy efficiency of the system increased by 4.0% and 5.7%, respectively. The net output power of the system increased from 23,988.5 W to 25,349.5 W. From Figure 7d, it can be obtained that as the anode inlet pressure increases, the ecological function E increases and the improvement potential IP decreases. Overall, increasing the anode inlet pressure can effectively improve the efficiency and output power of the system.

### 4.3. Effect of Cathode and Anode Stoichiometry

**Figure 8:**
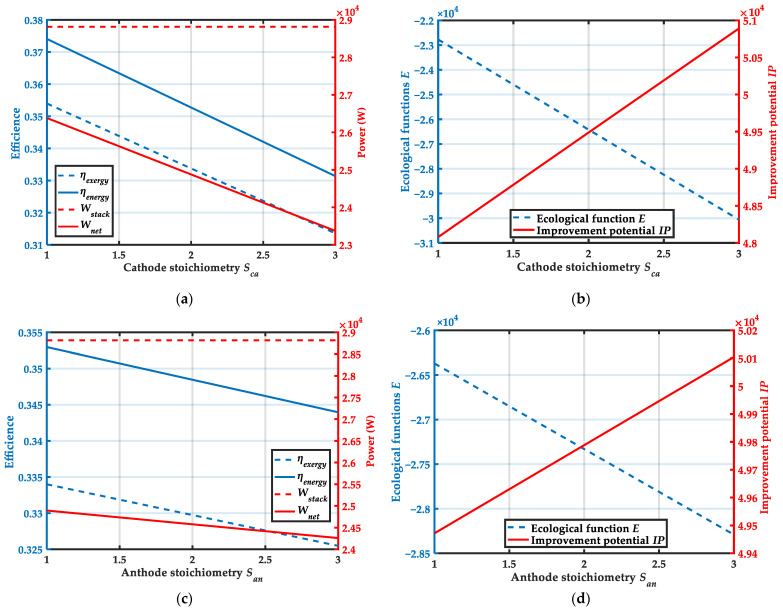
Variation trends of system performance with cathode and anode stoichiometry: (**a**) Variation trends of $\eta_{energy}$, $\eta_{exergy}$, $W_{stack}$ and $W_{net}$ with cathode stoichiometry $S_{ca}$ (**b**) Variation trends of ecological function E and improvement potential IP with cathode stoichiometry $S_{ca}$. (**c**) Variation trends of $\eta_{energy}$, $\eta_{exergy}$, $W_{stack}$ and $W_{net}$ with anode stoichiometry $S_{an}$; (**d**) Variation trends of ecological function E and improvement potential IP with anode stoichiometry $S_{an}$.

Figure 8 shows the variation trends of system performance with cathode and anode stoichiometry. From Figure 8a, it can be obtained that the increase in the cathode stoichiometry has little effect on the output power of the stack. When the cathode stoichiometry increases from 1 to 3, the energy efficiency and exergy efficiency of system decreased from 37.4% and 35.4% to 33.1% and 31.4%, respectively. The net output power of the system decreases from 26,382.1 W to 23,372.1 W. It can be obtained from Figure 8c that the increase in anode stoichiometry has no effect on the output power of the stack. As the anode stoichiometry increases, the energy efficiency decreases from 35.3% to 34.4%, the exergy efficiency reduces from 33.4% to 32.6%, and the net system output power decreases from 24,893.2 W to 24,258.3W. According to Figure 8b,d, it can be observed that when the stoichiometry increases, the ecological function E decreases and the improvement potential IP increases. The stoichiometry directly affects the mass flow rate of the gas. When the gas flow rate boosts, the output power of the HT-PEMFC stack has no change, but increases the power consumption of the ancillary equipment. As a result, it can be concluded that increasing the stoichiometry does not improve the output performance of the system. The cathode stoichiometry and anode stoichiometry should be as low as possible while ensuring adequate reaction of the gas in the stack.

## 5. Conclusions

In this paper, a thermodynamic model of a vehicular HT-PEMFC system using PA doped PBI membrane based on Matlab is developed, which includes the HT-PEMFC stack and the ancillary equipments to maintain the operating conditions of the stack. The system model can predict the temperature and pressure distribution of the fluid in each state, as well as the energy loss and exergy loss of each component in the system. Through thermodynamic analysis of the system the main conclusions can be drawn:

The efficiency of hydrogen use can be effectively improved by using a hydrogen compressor. At the current density J=0.8 A cm^2^, heating the inlet gases by coolant can improve the system energy efficiency by 3.15%. Meanwhile, the energy recovery of cathode exhaust gas using a turbine can make the system energy efficiency improved by 2.67%. The exergy loss of the HT-PEMFC stack and heat exchanger is relatively large. To improve the system output performance, on the one hand, the waste heat loss at the heat exchanger should be reduced, and waste heat utilization can be improved by means of cogeneration, etc. On the other hand, the energy conversion efficiency at the HT-PEMFC stack should be improved to reduce the exergy loss. The ecological performance of the system is better at lower current densities region. However, vehicular HT-PEMFC systems require higher power density stacks so as to facilitate installation and save space. Therefore, the operating current density of the HT-PEMFC stack should be carefully designed according to the specific requirements of the vehicle power system in practical applications. When the inlet gas temperature is 406.1 K, the energy efficiency, exergy efficiency and net output power of the system reach the maximum. And if the inlet gas temperature is too high, the power consumption of auxiliary equipment will increase. The ecological performance of the system is better in the relatively low range of the cathode inlet pressure. And the system output performance improves with the increase of anode inlet pressure. In addition, the cathode and anode stoichiometry should be reduced as much as possible to improve the system output performance on the basis of ensuring sufficient gas reaction in the stack.

## Figures and Tables

**Table 1:** Operating and design parameters of a vehicular HT-PEMFC system.

Component	Parameters	Values
HT-PEMFC stack	Number of single cells N	250
	Effective working area A	$300\;{\mathrm{cm}}^{2}$ [59]
	$\mathrm{Anode}\;\mathrm{stoichiometry}\;S_{an}$	1.05 [62]
	$\mathrm{Cathode}\;\mathrm{stoichiometry}\;S_{ca}$	2 [62]
	$\mathrm{Anode}\;\mathrm{inlet}\;\mathrm{pressure}\;p_{11}$	2 atm
	$\mathrm{Cathode}\;\mathrm{inlet}\;\mathrm{pressure}\;p_{4}$	2 atm
	$\mathrm{Inlet}\;\mathrm{temperature}\;T_{in}$	403 K [42]
	Relative humidity RH	7.6% [42]
	current density J	$0–2\;{A\;\mathrm{cm}}^{-2}$ [42]
Compressors	$\mathrm{Efficiency}\;\eta_{C}$	55% [62]
Coolant pump	$\mathrm{Efficiency}\;\eta_{CP}$	55%
Turbine	$\mathrm{Efficiency}\;\eta_{TUR}$	65%

**Table 2:** Power of each component at different current densities.

Components (Power: W)	0.2	0.4	0.6	0.8	1.0	1.2	1.4
HT-PEMFC stack	9156.9	16,588.3	23,106.4	28,815.2	33,675.9	37,532.9	40,045.9
AC	−1125.8	−2251.6	−3377.4	−4503.2	−5629.1	−6754.9	−7880.7
HC	−2.4	−4.9	−7.3	−9.7	−12.2	−14.6	−17.0
CHE	−154.1	−308.1	−462.2	−616.3	−770.3	−924.4	−1078.4
AHE	−32.3	−64.6	−97.0	−129.3	−161.6	−193.9	−226.3
CP	−115.3	−254.1	−405.3	−567.6	−741.3	−928.7	−11,345
TUR	472.0	944.0	1416.0	1887.9	2359.9	2831.9	3303.9

**Table 3:** Exergy loss expressions for each component and exergy loss distribution at current density $J=0.8\;{A\;\mathrm{cm}}^{2}$.

Components (Exergy Loss: W)	Exergy Loss Expressions	Values (*J* = 0.8)
HT-PEMFC stack	$E_{x,4}+E_{x,11}+E_{x,15}-E_{x,5}-E_{x,12}-E_{x,16}-W_{stack}$	35,057.3
AH	$E_{x,19}-(E_{x,11}-E_{x,10})$	269.6
CH	$E_{x,18}-(E_{x,4}-E_{x,3})$	814.7
HC	$W_{HC}-(E_{x,13}-E_{x,12})$	8.5
AC	$W_{AC}-(E_{x,2}-E_{x,1})$	1709.2
AHE and CHE	$(E_{x,16}-E_{x,17}+W_{AHE}+W_{CHE})-(E_{x,10}-E_{x,9})-(E_{x,3}-E_{x,2})$	11,774.3
CP	$W_{CP}-(E_{x,15}-E_{x,14})$	345.2
TUR	$(E_{x,6}-E_{x,7})-W_{TUR}$	535.2
Hydrogen-mixing	$E_{x,8}+E_{x,13}-E_{x,9}$	9.5
Water knock	$E_{x,5}-E_{x,6}$	772.1

## Data Availability

Not applicable.
